# Supplementary material for: Cost of surgical treatment for ulnar nerve entrapment in Finland, 2011–2015: a registry-based cost description study
Source: BMJ Open. 2023 Jun 1;13(6):e068964. doi: 10.1136/bmjopen-2022-068964 (PMC10255115; doi:10.1136/bmjopen-2022-068964)
Supplement: Supplementary data [file bmjopen-2022-068964supp001.pdf]

Supplementary Table 1.

	2011		2012		2013		2014		2015	
Hospital district	population, N	DRG price, €	population, N	DRG price, €	population, N	DRG price, €	population, N	DRG price, €	population, N	DRG price, €
South Karelia	132527	1330	132355	1839	132252	1822	131764	1860	131155	1940
Southern Ostrobothnia	198671	1525	198944	1654	198831	1878	198242	2038	197371	2043
Southern Savonia	105450	1472	104803	1552	104407	1552	103873	1552	103278	1552
Helsinki and Uusimaa	1545034	1150	1562796	1365	1581450	1345	1599390	1240	1616321	1320
Kymenlaakso	174827	1582	174466	1629	173864	1629	172908	1629	171778	1629
Pirkanmaa							524447	1575	526941	1369
Northern Ostrobothnia							405635	1702	407160	1730
Northern Savonia	248130	981	248233	981	248430	981	248407	1105	248129	1105
Satakunta	225302	820	224934	1330	224556	830	223983	720	222957	600
Vaasa	167489	1153	168111	1234	168848	1678	169652	1748	170212	1445
	population-weighted average, €									
	1182		1386		1376		1409		1399	

Population-weighted averages of DRG008O prices from ten hospital districts in Finland, using the DRG prices valid between 2011 and 2015. Prices have not been adjusted for inflation.
