# Supplementary material for: Cost of surgical treatment for ulnar nerve entrapment in Finland, 2011–2015: a registry-based cost description study
Source: BMJ Open. 2023 Jun 1;13(6):e068964. doi: 10.1136/bmjopen-2022-068964 (PMC10255115; doi:10.1136/bmjopen-2022-068964)
Supplement: Author's manuscript [file bmjopen-2022-068964.draft_revisions.pdf]

BMJ Open is committed to open peer review. As part of this commitment we make the peer review history of every article we publish publicly available.

When an article is published we post the peer reviewers' comments and the authors' responses online. We also post the versions of the paper that were used during peer review. These are the versions that the peer review comments apply to.

The versions of the paper that follow are the versions that were submitted during the peer review process. They are not the versions of record or the final published versions. They should not be cited or distributed as the published version of this manuscript.

BMJ Open is an open access journal and the full, final, typeset and author-corrected version of record of the manuscript is available on our site with no access controls, subscription charges or pay-per-view fees (http://bmjopen.bmj.com).

If you have any questions on BMJ Open's open peer review process please email info.bmjopen@bmj.com

BMJ Open

Cost of surgical treatment for ulnar nerve entrapment in Finland in 2011–2015.

Journal:	*BMJ Open*
Manuscript ID	bmjopen-2022-068964
Article Type:	Original research
Date Submitted by the Author:	12-Oct-2022

Complete List of Authors:	Hannula, Aarni; HUS Helsinki University Hospital, Department of Hand Surgery<br>Miettinen, Laura; Helsinki University Hospital and University of Helsinki, Department of Hand Surgery<br>Lampainen, Kaisa; Helsinki University Hospital and University of Helsinki, Department of Hand Surgery<br>Ryhänen, Jorma; Helsinki University Hospital and University of Helsinki, Department of Hand Surgery<br>Torkki, Paulus; Helsingin Yliopisto, Department of Public Health<br>Hulkkonen, Sina; Helsinki University Hospital and University of Helsinki, Department of Hand Surgery

Keywords:	Hand & wrist < ORTHOPAEDIC & TRAUMA SURGERY, Neurosurgery < SURGERY, Health economics < HEALTH SERVICES ADMINISTRATION & MANAGEMENT

SCHOLARONE™
Manuscripts

I, the Submitting Author has the right to grant and does grant on behalf of all authors of the Work (as defined in the below author licence), an exclusive licence and/or a non-exclusive licence for contributions from authors who are: i) UK Crown employees; ii) where BMJ has agreed a CC-BY licence shall apply, and/or iii) in accordance with the terms applicable for US Federal Government officers or employees acting as part of their official duties; on a worldwide, perpetual, irrevocable, royalty-free basis to BMJ Publishing Group Ltd ("BMJ") its licensees and where the relevant Journal is co-owned by BMJ to the co-owners of the Journal, to publish the Work in this journal and any other BMJ products and to exploit all rights, as set out in our licence.

The Submitting Author accepts and understands that any supply made under these terms is made by BMJ to the Submitting Author unless you are acting as an employee on behalf of your employer or a postgraduate student of an affiliated institution which is paying any applicable article publishing charge ("APC") for Open Access articles. Where the Submitting Author wishes to make the Work available on an Open Access basis (and intends to pay the relevant APC), the terms of reuse of such Open Access shall be governed by a Creative Commons licence – details of these licences and which Creative Commons licence will apply to this Work are set out in our licence referred to above.

Other than as permitted in any relevant BMJ Author's Self Archiving Policies, I confirm this Work has not been accepted for publication elsewhere, is not being considered for publication elsewhere and does not duplicate material already published. I confirm all authors consent to publication of this Work and authorise the granting of this licence.

For peer review only

Cost of surgical treatment for ulnar nerve entrapment in Finland in 2011–2015.

Hannula, Aarni, MD;[1] Miettinen, Laura, MD;[1] Lampainen, Kaisa, MD;[1] Ryhänen, Jorma, MD, PhD;[1] Torkki, Paulus, PhD;[2] Hulkkonen, Sina, MD, PhD.[1]

[1] Department of Hand Surgery, Helsinki University Hospital and University of Helsinki, Finland, Töölö hospital, Topeliuksenkatu 5, Helsinki.

[2] Department of Public Health, Faculty of Medicine, University of Helsinki, Finland, Tukholmankatu 8 B, Helsinki.

Correspondence to Hannula Aarni, Department of Hand Surgery, Helsinki University Hospital and University of Helsinki, Finland.

E-mail: aarni.hannula@helsinki.fi

Address: Topeliuksenkatu 5B, 00260, Helsinki, Finland

Word count: 2274

For peer review only

ABSTRACT

Objectives

The aim of this study was to evaluate the cost of surgical treatment for ulnar nerve entrapment (UNE) borne by the public sector in Finland.

Design

Cost description

Setting

Primary and secondary care in whole of Finland

Participants

We identified all patients diagnosed with primary UNE in the whole population of Finland between 2011 and 2015 from the Care Register for Health Care. Surgery-related costs were based on the diagnosis-related group prices from 2011 and 2017, provided by the Finnish Institute for Health and Welfare. The number of reimbursed sick leaves within the two years after diagnosis was collected from The Social Insurance Institution of Finland. We also combined an approximation of the costs related to referral in primary health care, diagnostic methods, and follow-up.

Interventions

Ulnar nerve release.

Primary and secondary outcome measures

Cost in 2015 euros.

Results

During our study period, approximately 1786 primary UNE diagnoses were made yearly, and 49% of patients received surgical treatment. Surgery-related cost averaged at EUR 1341 and reimbursed sick leaves at EUR 952 during this period. The annual cost of surgical treatment for UNE borne by the public sector in Finland varied between EUR 3082 and 3213.

For peer review only

Conclusions

The average cost of surgical treatment for UNE in Finland was EUR 3140 between 2011 and 2015.

Key terms

cost analysis; cost description; entrapment neuropathy; cubital tunnel syndrome

For peer review only

ARTICLE SUMMARY

Strengths and limitations of this study

Our population consist of the whole of Finnish population and not a subgroup of beneficiaries. The Finnish Care Register for Health Care and data provided by the Social Insurance Institution of Finland made it possible to accurately estimate the cost of reimbursed sick leave paid by the public sector.

We did not compare costs or efficacy between different medical interventions, and our results cannot be used directly in healthcare decision-making. This study can be seen as a reference baseline for further cost studies and efficiency estimations.

The number and types of healthcare contacts, in addition to the actual surgery and routinely done electroneuromyography, is not based on exact research material but the expert opinion of routinely done practice in Finland.

For peer review only

INTRODUCTION

The ageing population and rising healthcare costs are increasing the need to prioritize efficient treatment strategies. To make an objective comparison of different treatment strategies, an estimate of the cost is as necessary to obtain as the health-related results of interventions. Compression neuropathies of the upper limbs are common conditions and are often surgically treated. However, studies examining the costs of ulnar nerve entrapment (UNE) or its surgical treatment is scarce. The aim of this study was to evaluate the costs of surgical treatment for UNE borne by the public sector in Finland between 2011 and 2015.

Ulnar nerve entrapment

UNE is the second most common compression neuropathy in the upper extremity [1]. The most common compression site is at the elbow, at the cubital tunnel, whereas compression in wrist in Guyon's canal is much less frequent [2]. A Finnish population-based study concluded that the average age-standardized incidence rate of UNE per 100,000 person years was 36.1 among men and 26.3 among women [3]. UNE most commonly affects working-aged people and its incidence peaks around late middle age in both sexes [3–6]. UNE can cause numbness, paresthesia and pain in the arm and hand following ulnar nerve distribution and weakness of the ulnar nerve innervated muscles. In Finland, the diagnosis is based on typical symptoms, clinical findings, and positive electroneuromyography (ENMG) findings.

Surgical treatment of UNE

Most patients with mild symptoms benefit from conservative treatment and can be treated without surgery [2]. Prevalent surgical techniques for cubital tunnel syndrome include local

For peer review only - http://bmjopen.bmj.com/site/about/guidelines.xhtml

decompression and anterior subcutaneous or submuscular transposition of the ulnar nerve. Medial epicondylectomy can be incorporated if needed. There seems to be no difference between the effectiveness of anterior transpositions and of simple decompression [1]. In Finland, the prevalent surgical technique is simple decompression with surgical block anaesthesia, and the procedure is most often performed as day surgery.

METHODS

Study registries

In Finland, every public and private healthcare provider is obligated by law to provide information on procedures and inpatient episodes to the Finnish Institute for Health and Welfare (FIHW) for administrative and research purposes. Patient-level data, including social security number, diagnoses, and the Nordic classification of surgical procedure (NCSP) codes are stored in the Care Register for Health Care – a national register of both public and private hospital data in Finland [7]. The diagnoses are coded according to the International Classification of Diagnoses (ICD). UNE diagnosis is coded G56.2 according to the tenth revision of ICD since 1996.

The Social Insurance Institution of Finland (Kela) keeps a register of the total amount paid in euros and the incidence of benefits and their distribution by region and population group. This comprises all social benefits, including reimbursed sick leaves and drugs. Kela compensates 70% of workers' average wage after ten days of sick leave, for up to 300 days [8]. We obtained permission from FIHW and Kela to study and publish from these registries. The study was approved by the Ethics Committee of Northern Ostrobothnia (ETTMK 107/2017).

For peer review only

For peer review only - http://bmjopen.bmj.com/site/about/guidelines.xhtml

Population

The study population consisted of the whole population of Finland between 2011 and 2015. The Care Register for Health Care provided the total number of patients diagnosed with UNE during the study period. We included those with the diagnosis code G56.2 according to the tenth revision of ICD. Diagnosis code G56 (mononeuropathies of the upper limb) were also defined as UNE if they had a concomitant G56.2 diagnosis. Only incident cases were included in the study to evaluate the costs of primary UNE. All patients with a concomitant diagnosis of carpal tunnel syndrome were excluded. Surgically treated patients were defined as those with NSCP code ACC53, ulnar nerve release, during the year of diagnosis or the following year.

Cost framework

To approximate the cost of the surgical treatment of UNE, we combined the cost of diagnosis and referral in primary health care, ENMG-study, the cost of outpatient visit and telephone follow-up in specialized care, the cost of surgery and its related costs, as well as the amount of euros reimbursed for sick leaves. All costs are expressed in 2015 euros, using the consumer price index [9].

Cost of surgery and reimbursed sick leaves

As the cost of surgery, we used the diagnosis related group (DRG) prices from 2011 and 2017 provided by FIHW. These prices are based on the financial data from all five university hospitals and five central hospitals in Finland. Prices for the missing years 2012–2015 were

For peer review only

linearly interpolated from these years. DRG prices comprise total cost of care, including overheads, but not patient fees. We validated these prices by conducting a survey on DRG prices among the financial services of ten hospital districts that used DRG pricing during the study period. In order to estimate the total cost of surgery based on our survey, we calculated the population-weighted averages from the DRG prices.

The DRG008O cost code (surgery of nervous system, other, short treatment, non-complicated) was used to estimate the operating costs of the cubital tunnel release. This includes day surgery nerve operations, except for the release of the carpal tunnel, which has its own DRG code.

The total amount of funds paid for sick leaves with primary diagnosis of UNE (with the same exclusion and inclusion as mentioned above) and the total amount of reimbursed days of sick leave, and their distribution by length (exceeding ten days) was acquired from Kela's register.

Approximated costs

For diagnosis and referral in the primary care, we counted one outpatient visit with a general practitioner. One ENMG study, used for the diagnosis of entrapment neuropathies was also calculated per patient. We counted one preoperative surgeon appointment and one follow-up telephone appointment in specialized care as this was the most usual arrangement in our experience. A visit to a nurse in primary care was counted as a cost for the removal of stitches.

The unit cost for an outpatient appointment with a general practitioner, surgeon, and an outpatient appointment with a nurse in primary care was provided by FIHW. For the unit cost of follow-up appointment by telephone and ENMG study, we used the unit costs of the largest

For peer review only - http://bmjopen.bmj.com/site/about/guidelines.xhtml

hospital district in Finland and its diagnostic center, as FIHW did not provide these unit costs.

Ethical approval declaration

We obtained permission from the Finnish Institute for Health and Welfare and The Social Insurance Institution of Finland to study and publish from these registries. The study was approved by the Ethics Committee of Northern Ostrobothnia (ETTMK 107/2017).

Patient and public involvement

Patients or the public were not involved in the design, or conduct, or reporting, or dissemination plans of our research

RESULTS

During our study period, 1786 (SD 106,9) new UNE diagnoses were made on average per year. Of the patients diagnosed with UNE, 49% (SD 2.7%) underwent surgical treatment. Figure 1 shows the number of newly diagnosed UNE patients who underwent surgery or received non-surgical treatment per year.

Figure 2 shows the total annual cost of surgical treatment of patients. During the years under study, the total cost per 100,000 population was EUR 57,567 to EUR 74,122. The cost of treatment varied between EUR 44,351 and EUR 56,630 per 100,000 people, and sick leave between EUR 13,216 and EUR 17,576 per 100,000 population.

Of surgically treated patients, 27% had sick leave exceeding ten days. The average length of

For peer review only - http://bmjopen.bmj.com/site/about/guidelines.xhtml

these sick leaves was 40 days. This resulted in an average of 13,000 reimbursed days of work annually, incurring an average total cost of EUR 840,000 per year and an average cost of EUR 952 per surgically treated patient.

Figure 3 shows the cost formation each year. The results of our validation survey are shown as supplementary material (Supplementary Table 1). There was no statistical difference between the mean of annual DRG prices from our survey and the interpolated prices provided by FIHW (t-test, p=0,42).

DISCUSSION

We approximated the amount of public funds used in the treatment chain of surgically treating one UNE patient to be EUR 3082–3213 (2015 currency) in Finland in 2011–2015. The cost of treatment did not rise during the study period.

Our study's strength is that it included the cost of primary health care and a nerve conduction examination, which are often excluded or left unclear. In addition, our study population represented the whole of the Finnish population, not only a subgroup such as Medicare beneficiaries. The register covers the whole of the Finnish population and has been shown to be from satisfactory to very good in terms of completeness and overall accuracy, though this has not been studied exclusively in compression neuropathies [7]. UNE diagnostics are reasonably uniform in Finland, as ENMG is required for diagnosis. In Finland, every citizen is entitled to sick leave with pay, healthcare services, and surgical treatment when needed. This ensures patients' livelihoods and prevents them returning to work prematurely.

For peer review only

The study also had some limitations. As mentioned before, it only examined one upper extremity and did not consider possible revision procedures. Moreover, we used the DRG008O price for day surgery. The cost associated with possible hospital stay in the ward after surgery is not reflected in our results. In our experience, patients are seldom monitored in the ward overnight. It is also possible that the sick leaves in this study include bilaterally operated patients' sick leaves if the other extremity was operated on within two years of the initial surgery, inflating the cost of reimbursements. Sick leaves lasting ten days or less are missing, as they are not registered by Kela. This sick leave period does not represent the cost from our perspective, but is nonetheless a cost to the employer, who is mandated to pay the employee's salary for this period.

Most often, post-operative pain is treated with a short regimen of non-steroidal anti-inflammatory drugs, and as the standard reimbursement for a pack of one hundred 400 mg ibuprofen tablets is under five euros, we excluded the cost of drug reimbursements as insignificant. In Finland, opioids are not commonly used for managing post-operative pain after minor surgical procedures, such as the standard procedure for UNE.

We did not estimate the cost of complications, as they are not expected to significantly increase overall costs. The contribution and cost impact of subsequent revisions and bilateral surgeries were not assessed in this study, as they could not be analyzed based on the data.

The cost of UNE and its treatment has previously mostly been examined in studies comparing the costs associated with different surgical techniques, as it seems that these are equally effective [1,10–12]. The direct surgical cost of open decompression of the cubital tunnel, based on Medicaid reimbursements in 2005–2012 was USD 878–1149 [13], which is slightly lower

For peer review only - http://bmjopen.bmj.com/site/about/guidelines.xhtml

than our DRG price. However, in the same study the hospital charges were many times higher than the Medicaid reimbursements, indicating a possible shortfall.

A cost analysis conducted as a part of a randomized controlled trial in the Netherlands calculated that 94% of costs were caused by loss of production, including non-paid work, which was monetarily valued. It reported a very low cost of treatment – only EUR 151 per patient [12].

In conclusion, we estimated that the cost of the surgical treatment chain of ulnar nerve entrapment was on average EUR 3140 in Finland in 2011–2015. Our estimate is of a similar magnitude to the estimates previously made in the US.

For peer review only

Author Contributions A.H. and L.M. contributed equally for this research. All authors contributed to the study design and revision. A.H., L.M., K.L. and S.H. contributed to the acquisition and analysis of the data. A.H. and L.M. drafted the article. All authors approved the article to be published and to be accountable for all aspects of the work.

Competing interests: The authors disclose receipt of the following financial support for the research, authorship, and/or publication of this article: This work was supported by the Finnish Medical Foundation grant for S.H., grant number 5302. In the past 36 months P.T. has received grants or contracts from Merck, AstraZeneca, Roche, Abbvie and Promedical, has received consulting fees from Nordlic Healthcare Ltd and has been in leadership or fiduciary role in Effectiveness Society (Vaikuttavuusseura ry) and Association for children and youths with disabilities, Promedical Ltd.

Funding: The authors disclose receipt of the following financial support for the research, authorship, and/or publication of this article: This work was supported by the Finnish Medical Foundation grant for S.H., grant number 5302.

Informed consent: Informed consent was not sought for the present study because this is a register study, and according to Finnish law no informed consent is mandatory.

Data sharing statement: Research permit granted by the Finnish Institute for Health and Welfare and The Social Insurance Institution of Finland to publish from their records does not allow to share the data to outside parties.

Ethics Approval

The study was approved by the Ethics Committee of Northern Ostrobothnia (ETTMK 107/2017).

For peer review only

For peer review only - http://bmjopen.bmj.com/site/about/guidelines.xhtml

References

1 Caliandro P, la Torre G, Padua R, *et al.* Treatment for ulnar neuropathy at the elbow. *Cochrane Database of Systematic Reviews* 2016;**2016**. doi:10.1002/14651858.CD006839.pub4

2 Susan E. Mackinnon, Christine B. Novak. Compression Neuropathies. In: Scott W. Wolfe et al., ed. *Green's Operative Hand Surgery E-Book: Expert Consult: Online and Print.* Philadelphia: : Churchill Livingstone 2011. 977–1014.

3 Hulkkonen S, Lampainen K, Auvinen J, *et al.* Incidence and operations of median, ulnar and radial entrapment neuropathies in Finland: a nationwide register study. *Journal of Hand Surgery: European Volume* 2020;**45**:226–30. doi:10.1177/1753193419886741

4 Mondelli M, Giannini F, Ballerini M, *et al.* Incidence of ulnar neuropathy at the elbow in the province of Siena (Italy). *J Neurol Sci* 2005;**234**:5–10. doi:10.1016/j.jns.2005.02.010

5 Latinovic R, Gulliford MC, Hughes RAC. Incidence of common compressive neuropathies in primary care. *J Neurol Neurosurg Psychiatry* 2006;**77**:263–5. doi:10.1136/jnnp.2005.066696

6 Osei DA, Groves AP, Bommarito K, *et al.* Cubital tunnel syndrome: Incidence and demographics in a national administrative database. *Neurosurgery* 2017;**80**:417–20. doi:10.1093/neuros/nyw061

7 Sund R. Quality of the Finnish Hospital Discharge Register: A systematic review. *Scand J Public Health* 2012;**40**:505–15. doi:10.1177/1403494812456637

8 Social insurance institution of Finland. Sickness allowances. 2020.https://www.kela.fi/web/en/sickness-allowance (accessed 9 May 2022).

9 Official Statistics of Finland (OSF). Consumer price index [e-publication]. 2022.Available at: http://www.stat.fi/til/khi/tau_en.html

10 Song JW, Chung KC, Prosser LA. Treatment of ulnar neuropathy at the elbow: Cost-utility analysis. *Journal of Hand Surgery* 2012;**37**:1617–29. doi:10.1016/j.jhsa.2012.05.012

11 Kazmers NH, Lazaris EL, Allen CM, *et al.* Comparison of Surgical Encounter Direct Costs for Three Methods of Cubital Tunnel Decompression. *Plast Reconstr Surg* 2019;**143**:503–10. doi:10.1097/PRS.0000000000005196

12 Bartels RHMA, Termeer EH, van der Wilt GJ, *et al.* Simple decompression or anterior subcutaneous transposition for ulnar neuropathy at the elbow: A cost-minimization analysis - Part 2. *Neurosurgery* 2005;**56**:531–6. doi:10.1227/01.NEU.0000154132.40212.6E

13 Law TY, Hubbard ZS, Chieng LO, *et al.* Trends in Open and Endoscopic Cubital Tunnel Release in the Medicare Patient Population. *Hand* 2017;**12**:408–12. doi:10.1177/1558944716679610

For peer review only - http://bmjopen.bmj.com/site/about/guidelines.xhtml

Figure legends

Figure 1. number of surgically and non-surgically treated patients

Figure 2. Total cost of surgically treated patients in Finland in 2015 euros

Figure 3. Cost components per patient in 2015 euros

For peer review only

For peer review only - http://bmjopen.bmj.com/site/about/guidelines.xhtml

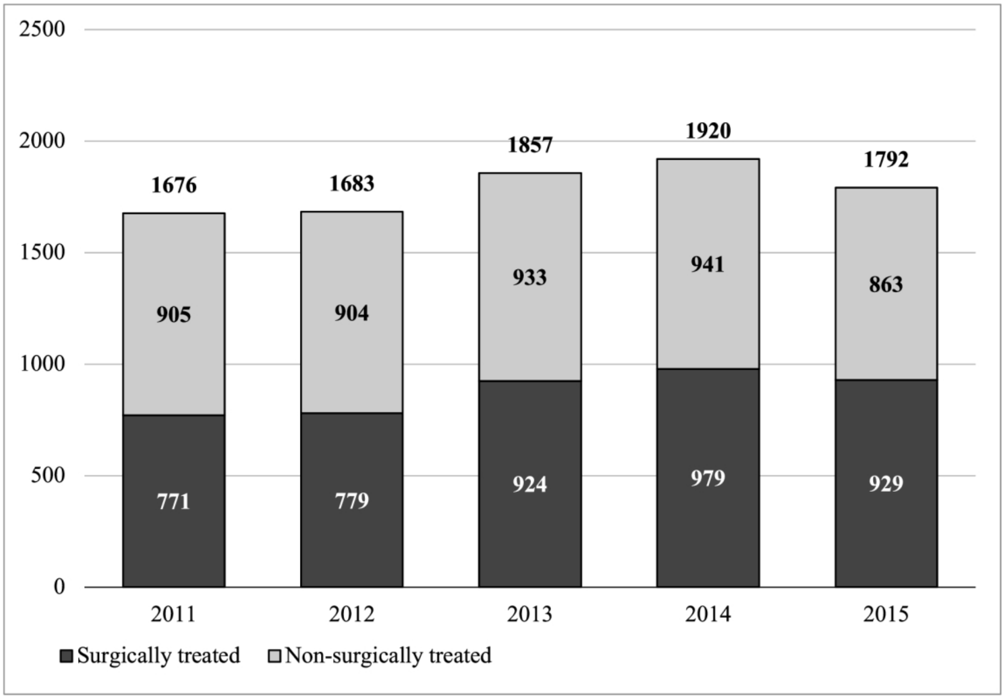

Figure 1. number of surgically and non-surgically treated patients

153x106mm (300 x 300 DPI)

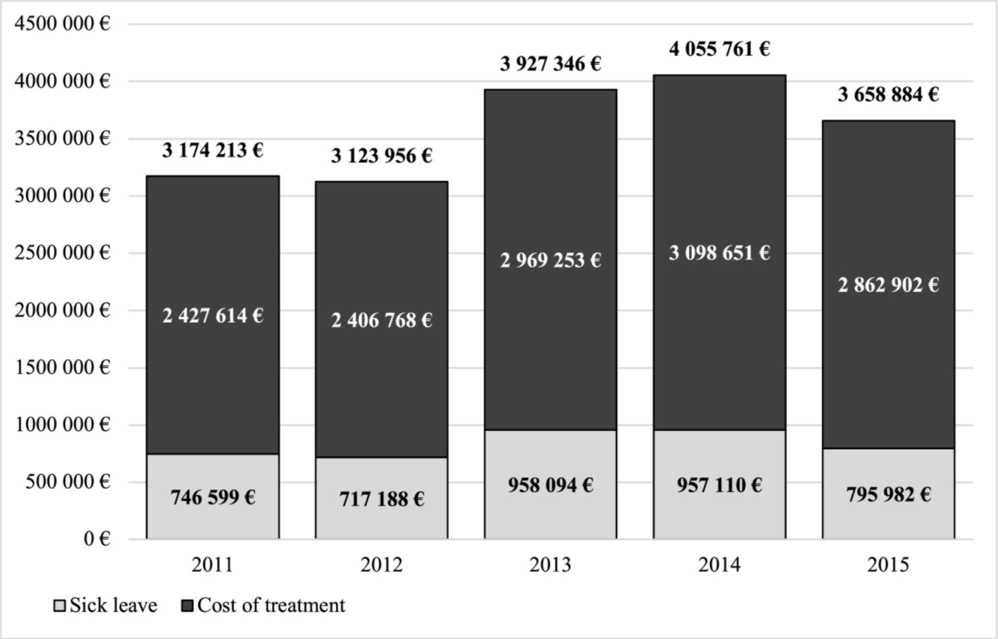

Figure 2. Total cost of surgically treated patients in Finland in 2015 euros

165x106mm (300 x 300 DPI)

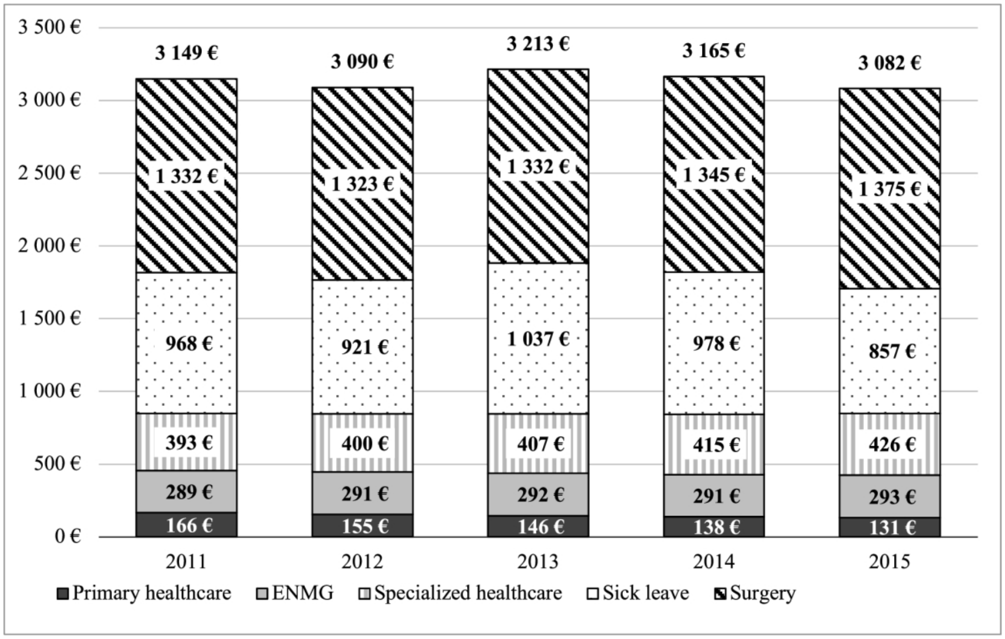

Figure 3. Cost components per patient in 2015 euros

168x106mm (300 x 300 DPI)

Supplementary Table 1.

	2011		2012		2013		2014		2015	
Hospital district	population, N	DRG price, €	population, N	DRG price, €	population, N	DRG price, €	population, N	DRG price, €	population, N	DRG price, €
South Karelia	132527	1330	132355	1839	132252	1822	131764	1860	131155	1940
Southern Ostrobothnia	198671	1525	198944	1654	198831	1878	198242	2038	197371	2043
Southern Savonia	105450	1472	104803	1552	104407	1552	103873	1552	103278	1552
Helsinki and Uusimaa	1545034	1150	1562796	1365	1581450	1345	1599390	1240	1616321	1320
Kymenlaakso	174827	1582	174466	1629	173864	1629	172908	1629	171778	1629
Pirkanmaa							524447	1575	526941	1369
Northern Ostrobothnia							405635	1702	407160	1730
Northern Savonia	248130	981	248233	981	248430	981	248407	1105	248129	1105
Satakunta	225302	820	224934	1330	224556	830	223983	720	222957	600
Vaasa	167489	1153	168111	1234	168848	1678	169652	1748	170212	1445
	population-weighted average, €									
	1182		1386		1376		1409		1399	

Population-weighted averages of DRG008O prices from ten hospital districts in Finland, using the DRG prices valid between 2011 and 2015. Prices have not been adjusted for inflation.

For peer review only

Reporting checklist for economic evaluation of health interventions.

Based on the CHEERS guidelines.

Instructions to authors

Complete this checklist by entering the page numbers from your manuscript where readers will find each of the items listed below.

Your article may not currently address all the items on the checklist. Please modify your text to include the missing information. If you are certain that an item does not apply, please write "n/a" and provide a short explanation.

Upload your completed checklist as an extra file when you submit to a journal.

In your methods section, say that you used the CHEERSreporting guidelines, and cite them as:

Husereau D, Drummond M, Petrou S, Carswell C, Moher D, Greenberg D, Augustovski F, Briggs AH, Mauskopf J, Loder E. Consolidated Health Economic Evaluation Reporting Standards (CHEERS) statement.

		Reporting Item	Page Number
**Title**			
	#1	Identify the study as an economic evaluation or use more specific terms such as "cost-effectiveness analysis", and describe the interventions compared.	1
**Abstract**			
	#2	Provide a structured summary of objectives, perspective, setting, methods (including study design and inputs), results (including base case and uncertainty analyses), and conclusions	2-3
**Introduction**			
Background and objectives	#3	Provide an explicit statement of the broader context for the study. Present the study question and its relevance for health policy or practice decisions	2-4
**Methods**			

For peer review only

Target population and subgroups	#4	Describe characteristics of the base case population and subgroups analysed, including why they were chosen.	7
Setting and location	#5	State relevant aspects of the system(s) in which the decision(s) need(s) to be made.	6
Study perspective	#6	Describe the perspective of the study and relate this to the costs being evaluated.	5
Comparators	#7	Describe the interventions or strategies being compared and state why they were chosen.	Not relevant
Time horizon	#8	State the time horizon(s) over which costs and consequences are being evaluated and say why appropriate.	7-8
Discount rate	#9	Report the choice of discount rate(s) used for costs and outcomes and say why appropriate	7
Choice of health outcomes	#10	Describe what outcomes were used as the measure(s) of benefit in the evaluation and their relevance for the type of analysis performed	Not relevant
Meaurement of effectiveness	#11a	Single study-based estimates: Describe fully the design features of the single effectiveness study and why the single study was a sufficient source of clinical effectiveness data	Not relevant
Measurement of effectiveness	#11b	Synthesis-based estimates: Describe fully the methods used for identification of included studies and synthesis of clinical effectiveness data	Not relevant
Measurement and valuation of preference based outcomes	#12	If applicable, describe the population and methods used to elicit preferences for outcomes.	Not relevant
**Estimating resources and costs **			
	#13a	Single study-based economic evaluation: Describe approaches used to estimate resource use associated with the alternative interventions. Describe primary or secondary research methods for valuing each resource item in terms of its unit cost. Describe any adjustments made to approximate to opportunity costs	Not relevant

Methods

For peer review only

Estimating resources and costs	#13b	Model-based economic evaluation: Describe approaches and data sources used to estimate resource use associated with model health states. Describe primary or secondary research methods for valuing each resource item in terms of its unit cost. Describe any adjustments made to approximate to opportunity costs.	Not relevant
Currency, price date, and conversion	#14	Report the dates of the estimated resource quantities and unit costs. Describe methods for adjusting estimated unit costs to the year of reported costs if necessary. Describe methods for converting costs into a common currency base and the exchange rate.	7-8
Choice of model	#15	Describe and give reasons for the specific type of decision analytical model used. Providing a figure to show model structure is strongly recommended.	Not relevant
Assumptions	#16	Describe all structural or other assumptions underpinning the decision-analytical model.	Not relevent
Analytical methods	#17	Describe all analytical methods supporting the evaluation. This could include methods for dealing with skewed, missing, or censored data; extrapolation methods; methods for pooling data; approaches to validate or make adjustments (such as half cycle corrections) to a model; and methods for handling population heterogeneity and uncertainty.	8
**Results**			
Study parameters	#18	Report the values, ranges, references, and, if used, probability distributions for all parameters. Report reasons or sources for distributions used to represent uncertainty where appropriate. Providing a table to show the input values is strongly recommended.	9-10
Incremental costs and outcomes	#19	For each intervention, report mean values for the main categories of estimated costs and outcomes of interest, as well as mean differences between the comparator groups. If applicable, report incremental cost-effectiveness ratios.	Not relevant
Characterising uncertainty	#20a	Single study-based economic evaluation: Describe the effects of sampling uncertainty for the estimated incremental cost and incremental effectiveness parameters, together with the impact of	Not relevant

For peer review only

		methodological assumptions (such as discount rate, study perspective).	
Characterising uncertainty	#20b	Model-based economic evaluation: Describe the effects on the results of uncertainty for all input parameters, and uncertainty related to the structure of the model and assumptions.	Not relevant
Characterising heterogeneity	#21	If applicable, report differences in costs, outcomes, or cost effectiveness that can be explained by variations between subgroups of patients with different baseline characteristics or other observed variability in effects that are not reducible by more information.	Not relevant
**Discussion**			
Study findings, limitations, generalisability, and current knowledge	#22	Summarise key study findings and describe how they support the conclusions reached. Discuss limitations and the generalisability of the findings and how the findings fit with current knowledge.	10-12
**Other**			
Source of funding	#23	Describe how the study was funded and the role of the funder in the identification, design, conduct, and reporting of the analysis. Describe other non-monetary sources of support	13
Conflict of interest	#24	Describe any potential for conflict of interest of study contributors in accordance with journal policy. In the absence of a journal policy, we recommend authors comply with International Committee of Medical Journal Editors recommendations	13

None The CHEERS checklist is distributed under the terms of the Creative Commons Attribution License CC-BY-NC. This checklist can be completed online using https://www.goodreports.org/, a tool made by the EQUATOR Network in collaboration with Penelope.ai

For peer review only

BMJ Open

Cost of surgical treatment for ulnar nerve entrapment in Finland, 2011–2015: a registry-based cost description study

Journal:	*BMJ Open*
Manuscript ID	bmjopen-2022-068964.R1
Article Type:	Original research
Date Submitted by the Author:	02-Mar-2023
Complete List of Authors:	Hannula, Aarni; HUS Helsinki University Hospital, Department of Hand Surgery<br>Miettinen, Laura; Helsinki University Hospital and University of Helsinki, Department of Hand Surgery<br>Lampainen, Kaisa; Helsinki University Hospital and University of Helsinki, Department of Hand Surgery<br>Ryhänen, Jorma; Helsinki University Hospital and University of Helsinki, Department of Hand Surgery<br>Torkki, Paulus; Helsingin Yliopisto, Department of Public Health<br>Hulkkonen, Sina; Helsinki University Hospital and University of Helsinki, Department of Hand Surgery
**Primary Subject Heading**:	Surgery
Secondary Subject Heading:	Health economics
Keywords:	Hand & wrist < ORTHOPAEDIC & TRAUMA SURGERY, Neurosurgery < SURGERY, Health economics < HEALTH SERVICES ADMINISTRATION & MANAGEMENT

SCHOLARONE™
Manuscripts

I, the Submitting Author has the right to grant and does grant on behalf of all authors of the Work (as defined in the below author licence), an exclusive licence and/or a non-exclusive licence for contributions from authors who are: i) UK Crown employees; ii) where BMJ has agreed a CC-BY licence shall apply, and/or iii) in accordance with the terms applicable for US Federal Government officers or employees acting as part of their official duties; on a worldwide, perpetual, irrevocable, royalty-free basis to BMJ Publishing Group Ltd ("BMJ") its licensees and where the relevant Journal is co-owned by BMJ to the co-owners of the Journal, to publish the Work in this journal and any other BMJ products and to exploit all rights, as set out in our licence.

The Submitting Author accepts and understands that any supply made under these terms is made by BMJ to the Submitting Author unless you are acting as an employee on behalf of your employer or a postgraduate student of an affiliated institution which is paying any applicable article publishing charge ("APC") for Open Access articles. Where the Submitting Author wishes to make the Work available on an Open Access basis (and intends to pay the relevant APC), the terms of reuse of such Open Access shall be governed by a Creative Commons licence – details of these licences and which Creative Commons licence will apply to this Work are set out in our licence referred to above.

Other than as permitted in any relevant BMJ Author's Self Archiving Policies, I confirm this Work has not been accepted for publication elsewhere, is not being considered for publication elsewhere and does not duplicate material already published. I confirm all authors consent to publication of this Work and authorise the granting of this licence.

For peer review only

Cost of surgical treatment for ulnar nerve entrapment in Finland, 2011–2015: a registry-based cost description study

Hannula, Aarni, MD;[1] Miettinen, Laura, MD;[1] Lampainen, Kaisa, MD;[1] Ryhänen, Jorma, MD, PhD;[1] Torkki, Paulus, PhD;[2] Hulkkonen, Sina, MD, PhD[1]

[1] Department of Hand Surgery, Helsinki University Hospital and University of Helsinki, Finland, Töölö hospital, Topeliuksenkatu 5, Helsinki, Finland

[2] Department of Public Health, Faculty of Medicine, University of Helsinki, Finland, Tukholmankatu 8 B, Helsinki, Finland

Correspondence to:

Hannula Aarni

Department of Hand Surgery, Helsinki University Hospital and University of Helsinki,

Topeliuksenkatu 5B, 00260, Helsinki, Finland

E-mail: aarni.hannula@helsinki.fi

Word count: 2167

For peer review only

ABSTRACT

Objectives

The aim of this study was to evaluate the cost of surgical treatment for primary ulnar nerve entrapment (UNE) borne by the public sector in Finland.

Design

Registry-based cost description study.

Setting

Primary and secondary care throughout Finland.

Participants

We identified all the patients diagnosed with primary UNE in the whole population of Finland from 2011 to 2015 from the Care Register for Health Care. From these patients, we identified those who had undergone ulnar nerve release during the year they were diagnosed or the following year.

Interventions

Open ulnar nerve release.

Outcome measures

The primary outcome measure was cost borne by the public sector in 2015 euros. The cost of surgery was based on the diagnosis-related group prices. We calculated the cost of a single visit to a primary care physician, an electroneuromyography examination, a preoperative visit to a hand surgeon and a follow-up appointment by telephone in specialized care for each patient. These unit costs were provided by the Finnish Institute for Health and Welfare and the same costs were used for each patient. We obtained the number of reimbursed sick days and the total amount reimbursed to each patient in euros within the two years after diagnosis was obtained from the Social Insurance Institution of Finland.

For peer review only

Results

During our study period, approximately 1786 primary UNE diagnoses were made yearly, and on average 876 (49%) of patients received surgical treatment annually. The surgery-related cost per patient averaged at EUR 1341 (43%) and reimbursed sick leaves at EUR 952 (30%) during this period. The annual cost of surgical treatment for UNE borne by the public sector in Finland varied between EUR 3082 and EUR 3213 per patient.

Conclusions

The average cost of surgical treatment for UNE in Finland was EUR 3140 per patient between 2011 and 2015.

Keywords

cost analysis; cost description; entrapment neuropathy; cubital tunnel syndrome

For peer review only

For peer review only - http://bmjopen.bmj.com/site/about/guidelines.xhtml

ARTICLE SUMMARY

Strengths and limitations of this study

- The data from the Finnish Care Register for Health Care and the Social Insurance Institution of Finland, both covering the whole population of Finland, made it possible to accurately estimate the cost of reimbursed sick leave paid by the public sector.
- The treatment protocol for ulnar nerve entrapment is uniform throughout Finland.
- We did not compare the costs or efficacy of different medical interventions, and our results cannot be used directly in healthcare decision-making.
- The number and types of healthcare contacts, in addition to the actual surgery and routine electroneuromyography, was not based on exact research material but on expert opinion of routine practice in Finland.

For peer review only

For peer review only - http://bmjopen.bmj.com/site/about/guidelines.xhtml

INTRODUCTION

The ageing population and rising healthcare costs are increasing the need to prioritize efficient treatment strategies. To enable an objective comparison of different treatment strategies, the costs must be estimated and the health-related results of interventions must be obtained. Compression neuropathies of the upper limbs are common conditions and are often surgically treated. However, studies examining the costs of ulnar nerve entrapment (UNE) or its surgical treatment are scarce. The aim of this study was to evaluate the costs of surgical treatment for UNE borne by the public sector in Finland between 2011 and 2015.

Ulnar nerve entrapment

UNE is the second most common compression neuropathy in the upper extremity [1]. The most common compression site is at the elbow, at the cubital tunnel, whereas compression in the wrist in Guyon's canal is much less frequent [2]. A Finnish population-based study concluded that the average age-standardized incidence rate of UNE per 100,000 person-years was 36.1 among men and 26.3 among women [3]. UNE most commonly affects working-aged people and its incidence peaks around late middle age in both sexes [3–6]. UNE can cause numbness, paraesthesia and pain in the arm and hand following ulnar nerve distribution and weakness of the ulnar nerve-innervated muscles. In Finland, diagnosis is based on typical symptoms, clinical findings, and positive electroneuromyography (ENMG) findings.

Surgical treatment of UNE

Most patients with mild symptoms benefit from conservative treatment and can be treated without surgery [2]. Prevalent surgical techniques for cubital tunnel syndrome include local

For peer review only - http://bmjopen.bmj.com/site/about/guidelines.xhtml

decompression and anterior subcutaneous or submuscular transposition of the ulnar nerve. Medial epicondylectomy can be incorporated if needed. The effectiveness of anterior transpositions and of simple decompression does not seem to differ [1].

In Finland, the prevalent surgical technique is simple decompression with surgical block anaesthesia, and the procedure is most often performed as day surgery. The endoscopic technique is exceedingly rare in Finland.

Complications are relatively rare. A meta-analysis estimated an average complication rate of 3% and revision rate of 2% after cubital tunnel release among all different surgical techniques. Simple decompression appears to be the safest option [7]. In a 2017 retrospective cohort study, simple decompression had a 3.8% (N=6) complication rate, of which 1.3 % (N=2) cases required surgery [8].

METHODS

Study registries

In Finland, every public and private healthcare provider is obligated by law to provide information on procedures and inpatient episodes to the Finnish Institute for Health and Welfare (FIHW) for administrative and research purposes. Patient-level data, including social security number, diagnoses, and the Nordic classification of surgical procedure (NCSP) codes are stored in the Care Register for Health Care – a national register of both public and private hospital data in Finland [9]. The diagnoses are coded according to the International Classification of Diagnoses (ICD). The code for UNE diagnosis is in accordance with the tenth

For peer review only

revision of ICD in 1996.

The Social Insurance Institution of Finland (Kela) keeps a register of the total amount paid in euros and of the incidence of benefits and their distribution by region and population group. This comprises all social benefits, including reimbursed sick leaves and drugs. Kela compensates 70% of workers' average pay after ten days of sick leave, for up to 300 days [10]. We obtained permission from the FIHW and Kela to study and publish from these registries. The study was approved by the Ethics Committee of Northern Ostrobothnia (ETTMK 107/2017).

Population

The study population was the whole population of Finland between 2011 and 2015. The Care Register for Health Care provided the total number of patients diagnosed with UNE during the study period. We included those with the diagnosis code G56.2 according to the tenth revision of ICD. Diagnosis code G56 (mononeuropathies of the upper limb) was also defined as UNE if the cases had a concomitant G56.2 diagnosis. Only incident cases were included in the study evaluating the costs of primary UNE. All patients with a concomitant diagnosis of carpal tunnel syndrome were excluded. Surgically treated patients were defined as those with the NSCP code ACC53, ulnar nerve release, which had occurred during the year of diagnosis or the following year.

Cost framework

To approximate the cost of the surgical treatment of UNE, we combined the costs of diagnosis

For peer review only - http://bmjopen.bmj.com/site/about/guidelines.xhtml

and referral in primary health care, ENMG examination, outpatient visits and telephone follow-up in specialized care, surgery and its related costs, and the amount of euros reimbursed for sick leaves. All costs are expressed in 2015 euros, using the consumer price index [11].

Cost of surgery and reimbursed sick leaves

As the cost of surgery, we used the diagnosis-related group (DRG) prices from 2011 and 2017 provided by the FIHW. These prices are based on the financial data from all five university hospitals and five central hospitals in Finland. The prices for the missing years of 2012–2015 were linearly interpolated from these years. The DRG prices comprised the total cost of care, including overheads but not patient fees. We validated these prices by conducting a survey on DRG prices among the financial services of ten hospital districts that used DRG pricing during the study period. To estimate the total cost of surgery based on our survey, we calculated the population-weighted averages from the DRG prices.

The DRG008O cost code (surgery of nervous system, other, short treatment, non-complicated) was used to estimate the operating costs of cubital tunnel release. This included day surgery nerve operations, excluding carpal tunnel release, which has its own DRG code.

The total amount of funds paid for sick leaves with a primary diagnosis of UNE (with the same exclusions and inclusions as mentioned above), and the total amount of reimbursed days of sick leave and their distribution by length (exceeding ten days) was acquired from Kela's register.

Approximated costs

For peer review only - http://bmjopen.bmj.com/site/about/guidelines.xhtml

For diagnosis and referral in primary care, we counted one outpatient visit with a general practitioner. One ENMG examination, used to diagnose of entrapment neuropathies was also calculated per patient. We counted one preoperative surgeon appointment and one follow-up telephone appointment in specialized care as this was the most usual arrangement in our experience. A visit to a nurse in primary care was counted as a cost for the removal of stitches.

The unit cost for an outpatient appointment with a general practitioner and a surgeon, and an outpatient appointment with a nurse in primary care was provided by the FIHW. For the unit cost of the follow-up appointment by telephone and the ENMG examination, we used the unit costs of the largest hospital district in Finland and its diagnostic centre, as the FIHW did not provide these unit costs.

Ethical approval

We obtained permission from the Finnish Institute for Health and Welfare and the Social Insurance Institution of Finland to study and publish from these registries. The study was approved by the Ethics Committee of Northern Ostrobothnia (ETTMK 107/2017).

Patient and public involvement

None.

RESULTS

For peer review only - http://bmjopen.bmj.com/site/about/guidelines.xhtml

During our study period, 1786 (SD 106.9) new UNE diagnoses were made on average per year. Of the patients diagnosed with UNE, 876 (49%, SD 95) underwent surgical treatment on average per year. Figure 1 shows the number of newly diagnosed UNE patients who underwent surgery or received non-surgical treatment per year.

Of the patients diagnosed with UNE, 5057 (56.6%) were men and 3871 (43.4%) women. The mean age was 50.84 (SD 14.95). Of the patients who underwent surgery, 2558 (58.4%) were men and 1824 were women (41.6%). Their mean age was 51.77 (SD 13.99).

Figure 2 shows the total annual cost of the surgical treatment of patients. During the years under study, the total cost per 100,000 population was EUR 57.567 to EUR 74.122. The cost of treatment varied between EUR 44,351 (76% of total costs) and EUR 56,630 (78%) per 100,000 people, and sick leave between EUR 13,216 (22%) and EUR 17,576 (24%) per 100,000 population.

Of the surgically treated patients, an average of 328 (37%) had more than ten sick leave days annually. The average length of these sick leaves was 40 days. This resulted in an average of 13,000 reimbursed days of work annually, incurring an average total cost of EUR 840,000 per year and an average cost of EUR 952 per surgically treated patient.

Figure 3 shows the cost formation per year. The results of our validation survey are shown as supplementary material (Supplementary Table 1). There was no statistical difference between the mean of the annual DRG prices in our survey and the interpolated prices provided by FIHW (t-test, p=0.42).

DISCUSSION

For peer review only - http://bmjopen.bmj.com/site/about/guidelines.xhtml

We approximated the amount of public funds used in the treatment chain to surgically treat one UNE patient as EUR 3082–3213 (2015 currency) in Finland in 2011–2015. The cost of treatment did not rise during the study period.

Our study's strength is that it included the cost of primary healthcare and a nerve conduction examination, which are often excluded or left unclear. In addition, our study population represented the whole of the Finnish population, not only a subgroup, such as Medicare beneficiaries. The register covers the entire Finnish population and has been shown to be from satisfactory to very good in terms of completeness and overall accuracy, though this has not been studied exclusively in compression neuropathies [9]. UNE diagnostics are reasonably uniform in Finland, as an ENMG is required for diagnosis. In Finland, every citizen is entitled to sick leave with pay, healthcare services, and surgical treatment when needed. This ensures patients' livelihoods and prevents them returning to work prematurely.

The study also has limitations. It only examined one upper extremity and did not consider possible revision procedures. Moreover, we used the DRG008O price for day surgery. The cost associated with possible hospital stay in the ward after surgery is not reflected in our results. In our experience, patients are seldom monitored in the ward overnight. It is also possible that the sick leaves in this study include bilaterally operated patients' sick leaves, that is, the other extremity was also operated on within two years of initial surgery. This would inflate the cost of reimbursements. The same is true for sick leaves resulting from the treatment of complications and possible revisions if the same ICD-10-code was used to prescribe the sick leaves. Sick leaves lasting ten days or less were missing, as they are not registered by Kela. This sick leave period does not represent the cost from our perspective, but is nonetheless a

For peer review only - http://bmjopen.bmj.com/site/about/guidelines.xhtml

cost to the employer, who is mandated to pay the employee's salary for this period.

Using a time trade-off survey and a decision analytic model, a 2012 study estimated that simple decompression, including possible complications and revisions, offers 2.76 quality-adjusted health years (QALY) of health benefit per treated patient in comparison to no treatment [12]. This is a substantial health benefit, considering the magnitude of the costs involved and the commonly used cost-effectiveness thresholds for acceptable cost of QALY in the reimbursement of pharmaceutics, for example GPD 20,000 – 30,000 by the National Institute of Health and Care Excellence in the United Kingdom [13]. In our study, we could not measure health benefits from the registries we used. A full economic evaluation, including both costs and benefits of all the relevant treatment strategies, could be a useful topic for future research.

Most often, post-operative pain is treated with a short regimen of non-steroidal anti-inflammatory drugs, and as the standard reimbursement for a pack of one hundred 400 mg ibuprofen tablets is under five euros, we excluded the cost of drug reimbursements as insignificant. In Finland, opioids are not commonly used for managing post-operative pain after minor surgical procedures such as the standard procedure for UNE.

We did not estimate the cost of complications, as they are not expected to significantly increase overall costs. Neither did we assess the contribution and cost impact of subsequent revisions and bilateral surgeries in this study, as they could not be analysed on the basis of the data.

The cost of UNE and its treatment has previously mostly been examined in studies comparing the costs associated with different surgical techniques, as it seems that these are equally effective [1,12,14,15]. The direct surgical cost of the open decompression of the cubital tunnel,

For peer review only

based on Medicaid reimbursements in 2005–2012 was USD 878–1149 [16], which is slightly lower than our DRG price. However, in the same study, the hospital charges were many times higher than the Medicaid reimbursements, indicating a possible shortfall.

A cost analysis conducted as part of a randomized controlled trial in the Netherlands calculated that 94% of costs were caused by loss of production, including non-paid work, which was monetarily valued. It reported a very low cost of treatment – only EUR 151 per patient [15].

In conclusion, we estimated that the cost of the surgical treatment chain of ulnar nerve entrapment was on average EUR 3140 in Finland in 2011–2015. This amount is similar to that of previously made estimates in the US.

For peer review only

Contributors: AH and LM contributed equally to this research. JR, PT and SH planned the study. AH, LM, KL, JR, PT and SH made a substantial contribution to the design of the work. AH, LM, KL and SH contributed to the acquisition and analysis of the data. AH and LM drafted the article. AH, LM, KL, JR, PT and SH revised the work for intellectual content. AH submitted the study. AH, LM, KL, JR, PT and SH approved the article for publication. AH, LM, KL, JR, PT and SH agreed to be accountable for all aspects of the work in ensuring that questions related to the accuracy or integrity of any part of the work are appropriately investigated and resolved.

Competing interests: The authors disclose receipt of the following financial support for the research, authorship, and/or publication of this article: In the past 36 months, PT has received grants or contracts from Merck, AstraZeneca, Roche, Abbvie and Promedical; consulting fees from Nordlic Healthcare Ltd and has acted in a leadership or fiduciary role in the Effectiveness Society (Vaikuttavuusseura ry) and the Association for children and youths with disabilities, Promedical Ltd. All other authors declare no competing interests.

Funding: The authors disclose receipt of the following financial support for the research, authorship, and/or publication of this article: This work was supported by the Finnish Medical Foundation grant for S.H., grant number 5302.

Data availability statement: The research permit granted by the Finnish Institute for Health and Welfare and the Social Insurance Institution of Finland to publish from their records does not allow sharing the data to external parties.

Ethics approval: The study was approved by the Ethics Committee of Northern Ostrobothnia (ETTMK 107/2017). We did not seek informed consent for the present study because it was a registry-based study, for which, according to Finnish law, informed consent is not required.

For peer review only

References

1 Caliandro P, la Torre G, Padua R, *et al.* Treatment for ulnar neuropathy at the elbow. *Cochrane Database of Systematic Reviews* 2016;**2016**. doi:10.1002/14651858.CD006839.pub4

2 Susan E. Mackinnon, Christine B. Novak. Compression Neuropathies. In: Scott W. Wolfe et al., ed. *Green's Operative Hand Surgery E-Book: Expert Consult: Online and Print.* Philadelphia: : Churchill Livingstone 2011. 977–1014.

3 Hulkkonen S, Lampainen K, Auvinen J, *et al.* Incidence and operations of median, ulnar and radial entrapment neuropathies in Finland: a nationwide register study. *Journal of Hand Surgery: European Volume* 2020;**45**:226–30. doi:10.1177/1753193419886741

4 Mondelli M, Giannini F, Ballerini M, *et al.* Incidence of ulnar neuropathy at the elbow in the province of Siena (Italy). *J Neurol Sci* 2005;**234**:5–10. doi:10.1016/j.jns.2005.02.010

5 Latinovic R, Gulliford MC, Hughes RAC. Incidence of common compressive neuropathies in primary care. *J Neurol Neurosurg Psychiatry* 2006;**77**:263–5. doi:10.1136/jnnp.2005.066696

6 Osei DA, Groves AP, Bommarito K, *et al.* Cubital tunnel syndrome: Incidence and demographics in a national administrative database. *Neurosurgery* 2017;**80**:417–20. doi:10.1093/neuros/nyw061

7 Wade RG, Griffiths TT, Flather R, *et al.* Safety and Outcomes of Different Surgical Techniques for Cubital Tunnel Decompression: A Systematic Review and Network Meta-analysis. *JAMA Netw Open* 2020;**3**. doi:10.1001/jamanetworkopen.2020.24352

8 Zhang D, Earp BE, Blazar P. Rates of Complications and Secondary Surgeries After In Situ Cubital Tunnel Release Compared With Ulnar Nerve Transposition: A Retrospective Review. *Journal of Hand Surgery* 2017;**42**. doi:10.1016/j.jhsa.2017.01.020

9 Sund R. Quality of the Finnish Hospital Discharge Register: A systematic review. *Scand J Public Health* 2012;**40**:505–15. doi:10.1177/1403494812456637

10 Social insurance institution of Finland. Sickness allowances. 2020.https://www.kela.fi/web/en/sickness-allowance (accessed 9 May 2022).

11 Official Statistics of Finland (OSF). Consumer price index [e-publication]. 2022.Available at: http://www.stat.fi/til/khi/tau_en.html

12 Song JW, Chung KC, Prosser LA. Treatment of ulnar neuropathy at the elbow: Cost-utility analysis. *Journal of Hand Surgery* 2012;**37**:1617–29. doi:10.1016/j.jhsa.2012.05.012

13 National Institute for Health and Care Excellence. Guide to the methods of technology appraisal 2013. *National Institute for Health and Care Excellence* 2013.

14 Kazmers NH, Lazaris EL, Allen CM, *et al.* Comparison of Surgical Encounter Direct Costs for Three Methods of Cubital Tunnel Decompression. *Plast Reconstr Surg* 2019;**143**:503–10. doi:10.1097/PRS.0000000000005196

15 Bartels RHMA, Termeer EH, van der Wilt GJ, *et al.* Simple decompression or anterior subcutaneous transposition for ulnar neuropathy at the elbow: A cost-minimization analysis - Part 2. *Neurosurgery* 2005;**56**:531–6. doi:10.1227/01.NEU.0000154132.40212.6E

For peer review only - http://bmjopen.bmj.com/site/about/guidelines.xhtml

16 Law TY, Hubbard ZS, Chieng LO, *et al.* Trends in Open and Endoscopic Cubital Tunnel Release in the Medicare Patient Population. *Hand* 2017;**12**:408–12. doi:10.1177/1558944716679610

For peer review only

For peer review only - http://bmjopen.bmj.com/site/about/guidelines.xhtml

Figure titles

Figure 1. Number of surgically and non-surgically treated patients

Figure 2. Total cost of surgically treated patients in Finland in 2015 euros

Figure 3. Cost components per patient in 2015 euros

For peer review only

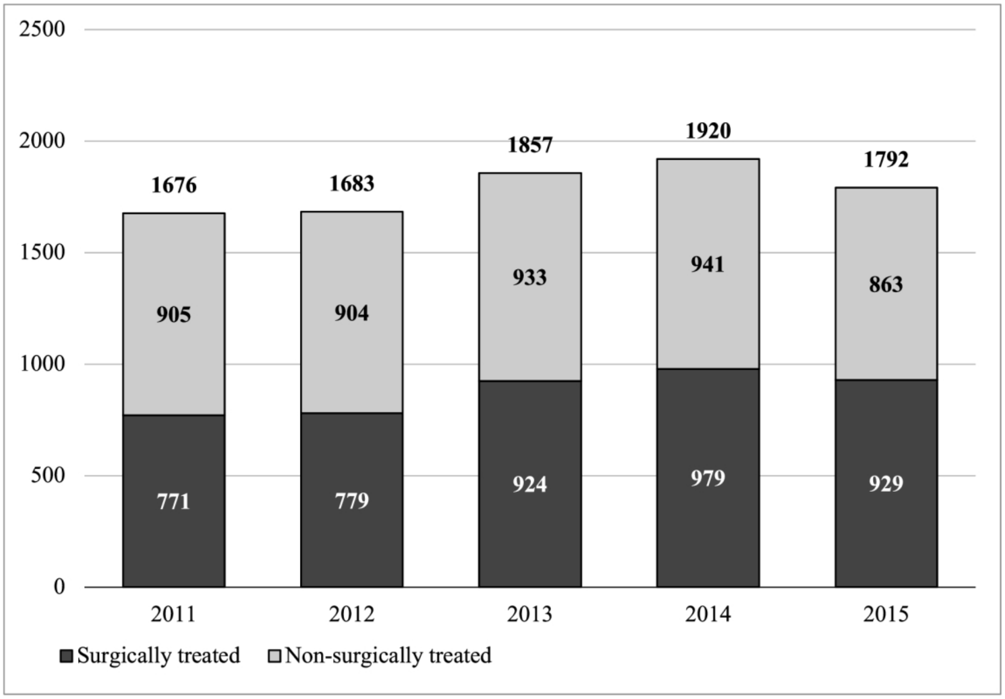

Figure 1. number of surgically and non-surgically treated patients

153x106mm (300 x 300 DPI)

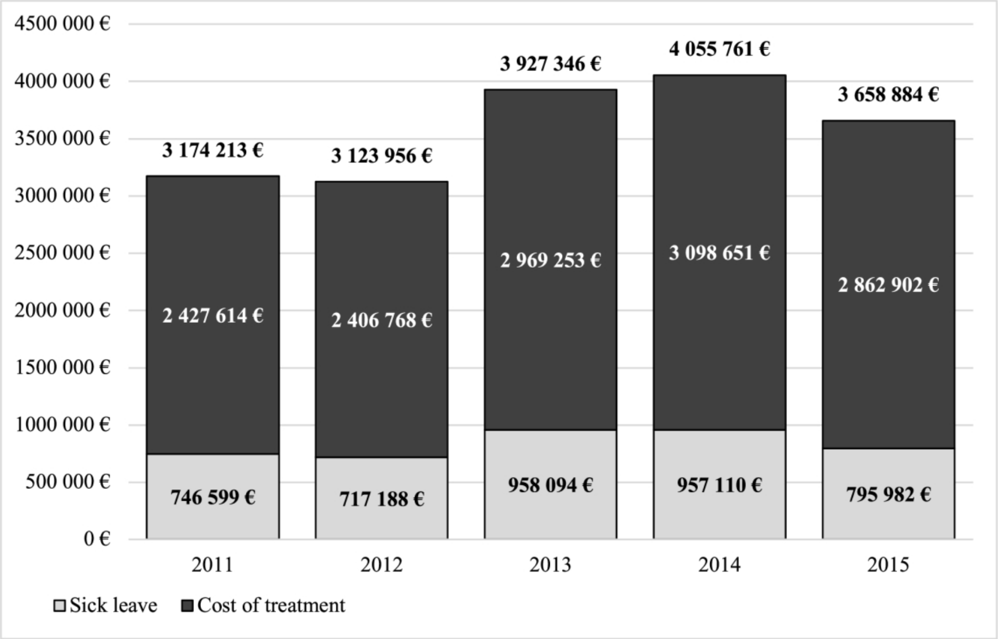

Figure 2. Total cost of surgically treated patients in Finland in 2015 euros

165x106mm (300 x 300 DPI)

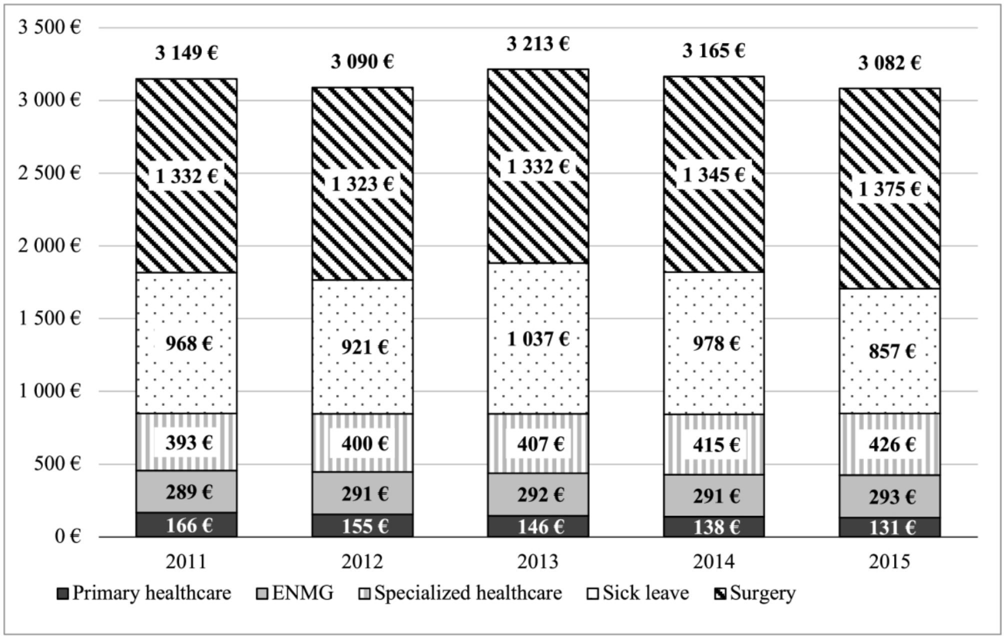

Figure 3. Cost components per patient in 2015 euros

168x106mm (300 x 300 DPI)

Supplementary Table 1.

	2011		2012		2013		2014		2015	
Hospital district	population, N	DRG price, €	population, N	DRG price, €	population, N	DRG price, €	population, N	DRG price, €	population, N	DRG price, €
South Karelia	132527	1330	132355	1839	132252	1822	131764	1860	131155	1940
Southern Ostrobothnia	198671	1525	198944	1654	198831	1878	198242	2038	197371	2043
Southern Savonia	105450	1472	104803	1552	104407	1552	103873	1552	103278	1552
Helsinki and Uusimaa	1545034	1150	1562796	1365	1581450	1345	1599390	1240	1616321	1320
Kymenlaakso	174827	1582	174466	1629	173864	1629	172908	1629	171778	1629
Pirkanmaa							524447	1575	526941	1369
Northern Ostrobothnia							405635	1702	407160	1730
Northern Savonia	248130	981	248233	981	248430	981	248407	1105	248129	1105
Satakunta	225302	820	224934	1330	224556	830	223983	720	222957	600
Vaasa	167489	1153	168111	1234	168848	1678	169652	1748	170212	1445
	population-weighted average, €									
	1182		1386		1376		1409		1399	

Population-weighted averages of DRG008O prices from ten hospital districts in Finland, using the DRG prices valid between 2011 and 2015. Prices have not been adjusted for inflation.

For peer review only

Reporting checklist for economic evaluation of health interventions.

Based on the CHEERS guidelines.

Instructions to authors

Complete this checklist by entering the page numbers from your manuscript where readers will find each of the items listed below.

Your article may not currently address all the items on the checklist. Please modify your text to include the missing information. If you are certain that an item does not apply, please write "n/a" and provide a short explanation.

Upload your completed checklist as an extra file when you submit to a journal.

In your methods section, say that you used the CHEERSreporting guidelines, and cite them as:

Husereau D, Drummond M, Petrou S, Carswell C, Moher D, Greenberg D, Augustovski F, Briggs AH, Mauskopf J, Loder E. Consolidated Health Economic Evaluation Reporting Standards (CHEERS) statement.

For peer review only

		Reporting Item	Page Number
**Title**			
	#1	Identify the study as an economic evaluation or use more specific terms such as “cost-effectiveness analysis”, and describe the interventions compared.	1
**Abstract**			
	#2	Provide a structured summary of objectives, perspective, setting, methods (including study design and inputs), results (including base case and uncertainty analyses), and conclusions	2-3
**Introduction**			
Background and objectives	#3	Provide an explicit statement of the broader context for the study. Present the study question and its relevance for health policy or practice decisions	5-6
**Methods**			

Target population and subgroups	#4	Describe characteristics of the base case population and subgroups analysed, including why they were chosen.	7
Setting and location	#5	State relevant aspects of the system(s) in which the decision(s) need(s) to be made.	6-7
Study perspective	#6	Describe the perspective of the study and relate this to the costs being evaluated.	5
Comparators	#7	Describe the interventions or strategies being compared and state why they were chosen.	Not relevant
Time horizon	#8	State the time horizon(s) over which costs and consequences are being evaluated and say why appropriate.	7--9
Discount rate	#9	Report the choice of discount rate(s) used for costs and outcomes and say why appropriate	8
Choice of health outcomes	#10	Describe what outcomes were used as the measure(s) of benefit in the evaluation and their relevance for the type of analysis performed	Not relevant
Meaurement of effectiveness	#11a	Single study-based estimates: Describe fully the design features of the single effectiveness study and why the single study was a sufficient source of clinical effectiveness data	Not relevant
Measurement of effectiveness	#11b	Synthesis-based estimates: Describe fully the methods used for identification of included studies and synthesis of clinical effectiveness data	Not relevant
Measurement and valuation of preference based outcomes	#12	If applicable, describe the population and methods used to elicit preferences for outcomes.	Not relevant
**Estimating resources and costs **			
	#13a	Single study-based economic evaluation: Describe approaches used to estimate resource use associated with the alternative interventions. Describe primary or secondary research methods for valuing each resource item in terms of its unit cost. Describe any adjustments made to approximate to opportunity costs	Not relevant

Methods

For peer review only

Estimating resources and costs	#13b	Model-based economic evaluation: Describe approaches and data sources used to estimate resource use associated with model health states. Describe primary or secondary research methods for valuing each resource item in terms of its unit cost. Describe any adjustments made to approximate to opportunity costs.	Not relevant
Currency, price date, and conversion	#14	Report the dates of the estimated resource quantities and unit costs. Describe methods for adjusting estimated unit costs to the year of reported costs if necessary. Describe methods for converting costs into a common currency base and the exchange rate.	7-9
Choice of model	#15	Describe and give reasons for the specific type of decision analytical model used. Providing a figure to show model structure is strongly recommended.	Not relevant
Assumptions	#16	Describe all structural or other assumptions underpinning the decision-analytical model.	Not relevent
Analytical methods	#17	Describe all analytical methods supporting the evaluation. This could include methods for dealing with skewed, missing, or censored data; extrapolation methods; methods for pooling data; approaches to validate or make adjustments (such as half cycle corrections) to a model; and methods for handling population heterogeneity and uncertainty.	7-9
**Results**			
Study parameters	#18	Report the values, ranges, references, and, if used, probability distributions for all parameters. Report reasons or sources for distributions used to represent uncertainty where appropriate. Providing a table to show the input values is strongly recommended.	10
Incremental costs and outcomes	#19	For each intervention, report mean values for the main categories of estimated costs and outcomes of interest, as well as mean differences between the comparator groups. If applicable, report incremental cost-effectiveness ratios.	Not relevant
Characterising uncertainty	#20a	Single study-based economic evaluation: Describe the effects of sampling uncertainty for the estimated incremental cost and incremental effectiveness parameters, together with the impact of	Not relevant

For peer review only

		methodological assumptions (such as discount rate, study perspective).	
Characterising uncertainty	#20b	Model-based economic evaluation: Describe the effects on the results of uncertainty for all input parameters, and uncertainty related to the structure of the model and assumptions.	Not relevant
Characterising heterogeneity	#21	If applicable, report differences in costs, outcomes, or cost effectiveness that can be explained by variations between subgroups of patients with different baseline characteristics or other observed variability in effects that are not reducible by more information.	Not relevant
**Discussion**			
Study findings, limitations, generalisability, and current knowledge	#22	Summarise key study findings and describe how they support the conclusions reached. Discuss limitations and the generalisability of the findings and how the findings fit with current knowledge.	11-13
**Other**			
Source of funding	#23	Describe how the study was funded and the role of the funder in the identification, design, conduct, and reporting of the analysis. Describe other non-monetary sources of support	14
Conflict of interest	#24	Describe any potential for conflict of interest of study contributors in accordance with journal policy. In the absence of a journal policy, we recommend authors comply with International Committee of Medical Journal Editors recommendations	14

None The CHEERS checklist is distributed under the terms of the Creative Commons Attribution License CC-BY-NC. This checklist can be completed online using https://www.goodreports.org/, a tool made by the EQUATOR Network in collaboration with Penelope.ai

For peer review only
